# Editorial: Tumor Systems Biology: How to Therapeutically Redirect Dysregulated Homeostasis in Tumor Systems (i.e., Anakoinosis)

**DOI:** 10.3389/fonc.2020.01675

**Published:** 2020-09-02

**Authors:** Albrecht Reichle, Daniel Heudobler, Christopher Gerner, Pan Pantziarka, Eugenio Martinelli, Ernst Holler, Francesca Corsi, Lina Ghibelli

**Affiliations:** ^1^Department of Internal Medicine III, Hematology and Oncology, University Hospital Regensburg, Regensburg, Germany; ^2^Faculty Chemistry, Institut for Analytical Chemistry, University Vienna, Vienna, Austria; ^3^The George Pantziarka TP53 Trust, London, United Kingdom; ^4^Anticancer Fund, Brussels, Belgium; ^5^Department of Electronic Engineering, University of Rome Tor Vergata, Rome, Italy; ^6^Department Biology, Universita' di Roma Tor Vergata, Rome, Italy; ^7^Department of Chemical Sciences and Technologies, Universita' di Roma Tor Vergata, Rome, Italy

**Keywords:** anakoinosis, reverse anakoinosis, tissue homeostasis, master modifiers of tumor tissues, biomodulatory drugs, chemoprevention, targeted tissue engineering, refractory metastatic cancer

During the course of metastatic tumor disease repopulation of cancer cells too often occurs, causing fatal relapses: cancer tissues “wounded” by cytotoxic or targeted therapies regenerate via the “phoenix rising” pathway that is mediated, counterintuitively, by the apoptotic cells themselves. This seems to be an intrinsic limit of such systemic tumor therapies, calling for a change of paradigm (Heudobler et al.): Anakoinosis, from the Grecian “communication,” represents a paradigmatic new pathophysiologic perspective describing aberrant homeostasis in cancer tissue for therapeutically redirecting dysbalanced homeostasis. Correcting tumor-associated homeostasis facilitates re-activation of lost tissue defenses. The use of compounds acting as “master modifiers” of gene expression or tissue homeostasis, enables even clinical continuous complete remission of otherwise untreatable, refractory metastatic neoplasia [Heudobler et al.; (1)]. To exemplify, “master modifiers” can induce complete hematologic remission in acute myelocytic leukemia, although p53 mutation is continuously detectable with a high mutational load (1).

It is emerging that continuous flux of information between different tissue components in tumors modulates gene expression, thereby, promoting tumor-suppressive pathways. Thus, epithelia can keep even cells with oncogene mutations in check (1). Cancer would thus only arise when tissues lose the homeostatic control on mutated cells.

The classes of substances discussed in this topic are “master modifiers” with even stimulatory activity, as they reprogram homeostatic pathways, at variance with classic pathway inhibitors as commonly used in oncology (Heudobler et al.).

## Novel Biomodulatory “Drug Families” As Master Modifiers of Tumor Tissues

A novel biomodulatory prospective is offered by the use of modulating RNAs for attenuating tumor proliferation or modifying immune response. Zharkov et al. pointed out the potential therapeutic importance of biomodulation with dsRNA for systemic tumor therapy. Like long non-coding RNA, small dsRNA represents a novel family of anakoinosis inducers. For therapeutic use, nanoparticles have to be doped with RNA.

Arsenic trioxide is routinely administered in patients with acute promyelocytic leukemia (APL). Dual biomodulation in combination with all-trans retinoic acid has curative potential in APL (2). Insofar, it is obvious to study arsenic minerals, in drug resistant solid tumors, such as in triple-negative breast cancer. In mice, a prolonged survival could be observed by inhibiting the activation of hypoxia-inducible factor-1α (HIF-1α) and NLRP3 inflammasomes with arsenic sulfide (As_4_S_4_) (Wang et al.). Here, it was shown that (As_4_S_4_) could be successfully delivered by nanoparticles, suggesting that they may act as promising carriers also for biomodulatory drugs with poor solubility.

López-Contreras et al. looked at indomethacin from a novel angle. The results indicate that indomethacin alters polyamine metabolism in NSCLC cells and enhances the effect of polyamine synthesis inhibitors. Interestingly, and this is typical for pro-anakoinotic processes, the effect of indomethacin *in vitro* varies depending on the basal metabolic fingerprint of each type of cancer cell (López-Contreras et al.).

Flavonoids, phytochemical compounds, are a huge class of natural polyphenols with multi-fold biomodulatory activities in tumor tissue. Antioxidant and anti-inflammatory activities of flavonoids are well-known. They modulate signaling pathways involved in cell survival, proliferation, differentiation, migration, angiogenesis, and hormone activities. Nobiletin, a flavonoid, was shown to inhibit cell viability in human renal carcinoma cells, thus possibly providing a novel promising biomodulatory component for the treatment of kidney cancer (Wei et al.).

## Overcoming Tumor Resistance By Exploiting Tumor Tissue's Plasticity

An important application field for biomodulation is overcoming drug resistance in refractory disease [(3); Heudobler et al.]. Genetic conditions in the tumor are prerequisite for tumor resistance, but also for consecutively developing non-selective mechanisms of resistance. Pharmacologic interventions exploiting the communication flux in tumor tissue and its metabolic plasticity are to a minor degree clinically exploited. Han et al. has shown that DLD-1 colon cancer cells with *BAX*/*BAK* double knockout are selectively resistant to a panel of FDA-approved drugs (27 out of 66), including etoposide. Drug resistance might be overcome via modification of the glycolytic pathway.

The methodology for overcoming tumor resistance toward classic tumor cell-directed therapy, i.e., anakoinosis, has been outlined in a review paper (Heudobler et al.). Again, the biomodulatory triple combination of azacitidine, pioglitazone, and all-trans retinoic could be presented as combination for overcoming azacitidine resistance, now in allogeneic relapse of AML. Although, the added transcriptional modulators show no monoactivity in AML, their pro-anakoinotic activity “normalizes” AML-associated homeostatic disbalances in a therapeutically meaningful way (Heudobler et al.). As already mentioned, outcome of biomodulatory therapies is strongly context-dependent and ranges in AML between continuous complete remission and stabilization of disease with peripheral blasts without need of transfusion [Kattner et al.; (1, 4, 5)].

## What Does Anakoinosis Accomplish?

Communication flux and homeostatic imbalances provide a pivotal starting point for designing appropriate biomodulatory therapy (Heudobler et al.). Comparing immune escape mechanisms in endometrial cancer with immune tolerance mechanisms occurring at the maternal–fetal interface, appreciates inter-cellular pathophysiologic processes and paves the way for anakoinosis-based therapies oriented at the current pathophysiologic state of tumor disease (Bruno et al.).

Chemoprevention seems to be an additional focus for anakoinosis. As clinically shown, anakoinosis has the potential for functionally marginalize acquired mutations, hopefully also germ line mutations. Strikingly, upon anakoinosis, AML blasts may re-acquire normal functions while maintaining an abnormal genetic pattern (1).

A novel expanding field for toxicological and environmental studies represents reverse anakoinosis. In analogy to pro-anakoinotic drug “cocktails” controlling metastatic and refractory tumor disease by a low-toxic biomodulatory approach, environmental compounds without suspicion of single drug toxicity might induce chronic disease and/or tumors by their concerted activity profile (Heudobler et al.).

Anakoinosis provides a methodology for targeted tissue engineering aimed at improving tumor outcome. As shown in multifold clinical trials, real life requirements may be successfully addressed by tissue modulation. Medical needs are given by frequently occurring therapeutic limitations, such as high patients age due to the age distribution of many neoplasia, age-associated comorbidity and/or refractivity of tumor disease.

## Author Contributions

AR and LG have written the manuscript. DH and FC have reviewed the manuscript. All authors contributed to the article and approved the submitted version.

## Conflict of Interest

The authors declare that the research was conducted in the absence of any commercial or financial relationships that could be construed as a potential conflict of interest.

## References

[1] HeudoblerDKlobuchSLükeFHahnJGrubeMKremersS Low-dose azacitidine, pioglitazone and all-trans retinoic acid versus standard-dose azacitidine in patients ≥ 60 years with acute myeloid leukemia refractory to standard induction chemotherapy (AMLSG 26-16/AML-ViVA): results of the safety run-in phase I. Blood. (2019) 134(Suppl_1):1382. 10.1182/blood-2019-12997737881883

[2] Cicconi L Fenaux P Kantarjian H Tallman M Sanz MA Lo-Coco F. Molecular remission as a therapeutic objective in acute promyelocytic leukemia. Leukemia. (2018) 32:1671–8. 10.1038/s41375-018-0219-5 30026570

[3] Heudobler D Rechenmacher M Lüke F Vogelhuber M Klobuch S Thomas S. Clinical Efficacy of a Novel Therapeutic Principle, Anakoinosis. Front Pharmacol. (2018) 9:1357. 10.3389/fphar.2018.01357 30546308 PMC6279883

[4] Thomas S Schelker R Klobuch S Zaiss S Troppmann M Rehli M. Biomodulatory therapy induces complete molecular remission in chemorefractory acute myeloid leukemia. Haematologica. (2015) 100:e4–6. 10.3324/haematol.2014.115055 25261094 PMC4281320

[5] Heudobler D Klobuch S Thomas S Hahn J Herr W Reichle A. Cutaneous leukemic infiltrates successfully treated with biomodulatory therapy in a rare case of therapy-related high risk MDS/AML. Front Pharmacol. (2018) 9:1279. 10.3389/fphar.2018.01279 30483125 PMC6243099

